# Immediate Dentine Sealing: Towards a Surface Science Perspective on an Undercharacterised Adhesive Interface

**DOI:** 10.3390/dj13120549

**Published:** 2025-11-21

**Authors:** Konstantinos Anastasiadis, Emmanouil-George Tzanakakis

**Affiliations:** 1 Independent Researcher, 15354 Gyka Nera, Greece; 2 Independent Researcher, 15561 Cholargos, Greece; tzanakak@dent.uoa.gr

**Keywords:** immediate dentine sealing, adhesive dentistry, bond strength, dentine bonding, interface characterisation, surface analysis techniques

## Abstract

**Background**: Immediate Dentine Sealing (IDS) is a well-established adhesive strategy that protects freshly cut dentine and enhances the clinical performance of indirect restorations. While its mechanical benefits are extensively documented, the surface morphology and chemical nature of the sealed dentine, particularly following provisionalisation and reactivation, remain under-characterised. Understanding this bonding substrate is critical for optimising adhesion and long-term outcomes. **Methods**: This narrative review synthesises the literature on the morphological and chemical features of dentine following IDS, focusing on the distinction between cross-sectional and surface-level characterisation, as well as the analytical techniques employed. **Results**: Most studies concentrate on internal bond strength and failure analysis, with only a limited subset incorporating surface-sensitive methods such as top-down SEM or optical non-contact profilometry. Quantitative and chemically resolved data on the reactivated dentine surface, the dentine surface after cleaning or abrasion, prior to cementation are scarce, and standardised analytical protocols are lacking. **Conclusions**: The bonding interface in IDS, namely the reactivated dentine surface, is underexplored. Future research should apply advanced, non-destructive techniques to characterise this clinically relevant substrate and guide the development of adhesive systems tailored to IDS-treated dentine.

## 1. Introduction

Immediate dentine sealing (IDS) refers to the application and polymerisation of a dentine bonding agent immediately after tooth preparation and prior to impression-taking or digital scanning. Introduced in 1999 and formalised in 2005 [1,2], the approach is biologically and biomechanically rational for enhancing the performance of indirect bonded restorations. This initial proposal also prompted critical yet constructive commentary from Perdigão, who acknowledged the biological rationale and clinical relevance of the IDS approach, while cautioning against potential limitations such as oxygen-inhibited layer stability and challenges in achieving reliable resin–resin bonding at a delayed stage [3]. In retrospect, these cautions anticipated lines of inquiry that remain incompletely addressed—such as the time-dependent behaviour of the oxygen-inhibited layer during provisionalisation, the reliability of delayed resin–resin co-polymerisation, and the heterogeneity of reactivation protocols—thereby helping to shape the present research agenda.

The clinical rationale underlying IDS is multifaceted. It includes the immediate protection of freshly exposed dentine against microbial and chemical insult [4], a reduction in pulpal sensitivity during the provisional phase [5], and the creation of an optimised hybridised substrate [6] under controlled moisture and haemostasis. Moreover, by enabling the formation of a mature adhesive layer prior to definitive cementation, IDS mitigates the risk of adhesive dilution and improves the wettability and flow dynamics of resin luting agents [7,8,9]. Collectively, these factors support the integration of IDS into contemporary adhesive workflows, particularly within indirect restorative protocols.

A substantial body of in vitro evidence has corroborated the mechanical advantages of IDS. Studies employing microtensile and microshear bond strength testing have consistently demonstrated superior adhesive performance relative to delayed dentine bonding, particularly when IDS is combined with an intermediate flowable resin layer [10,11,12]. In parallel, fracture analysis using scanning electron microscopy (SEM) has confirmed the formation of continuous hybrid layers and cohesive failure modes within the cement, suggesting stable long-term adhesion [13]. These findings have been synthesised in both systematic reviews and narrative appraisals [8,9,14], which generally support the efficacy of IDS in enhancing the durability and marginal integrity of indirect restorations. Moreover, preliminary clinical evidence has emerged, indicating favourable survival and complication rates in long-term IDS-treated restorations [15,16]. For clarity, a distinction is drawn between cross-sectional analyses of the adhesive interface (e.g., microtensile/microshear testing, fracture-mode analysis and cross-sectional SEM) and surface-level characterisation of the reactivated dentine surface immediately prior to cementation (topography, chemistry and wettability). Whereas previous summaries have primarily synthesised mechanical outcomes and cross-sectional observations, the present review focuses on the pre-cementation surface itself, mapping the limited surface-sensitive evidence and outlining minimal reporting considerations to support reproducible characterisation.

Notwithstanding this growing body of evidence, the literature remains largely focused on mechanical outcomes or cross-sectional analyses of bonded interfaces. As such, the sealed dentine surface itself, namely the substrate that directly receives the luting agent at the time of definitive restoration, remains insufficiently characterised from a morphological, elemental, and molecular standpoint, as well as to the surface modifications that may arise from common pre-cementation protocols, including air abrasion, diamond bur roughening, and prophylactic pumice cleaning [17]. Moreover, the potential ramifications of such alterations for the quality, longevity, and predictability of the final adhesive interface remain partially elucidated [18]. To our knowledge, no systematic review has synthesised the available evidence regarding the physicochemical nature of sealed dentine, nor has any scholarly work comprehensively outlined the analytical approaches, such as scanning electron microscopy (SEM), Fourier-transform infrared spectroscopy (FTIR), Raman microspectroscopy, X-ray photoelectron spectroscopy (XPS), Atomic force microscopy (AFM), time-of-flight secondary ion mass spectrometry (ToF-SIMS), or optical non-contact profilometry, that may be employed to characterise this clinically significant substrate.

Accordingly, the present review seeks to critically appraise the current evidence on the morphological and molecular characteristics of sealed dentine surfaces following IDS and to identify promising directions for future research based on available or underutilised characterisation techniques.

For clarity, the term reactivated dentine surface refers to the IDS-treated dentine immediately prior to cementation, following provisionalisation and mechanical/chemical reactivation.

## 2. Materials and Methods

This narrative review was conducted to investigate whether the sealed dentine surface following Immediate Dentine Sealing (IDS) has been morphologically or chemically characterised at various clinical stages, particularly in its final pre-cementation state. The primary research question was: “*Is there a documented characterisation of the dentine surface, previously treated with IDS, at the point where it has undergone surface reactivation and is ready to receive adhesive luting?*”

A comprehensive search was performed using PubMed and Google Scholar as the primary electronic databases. The search strategy included a combination of the following keywords: “*immediate dentine sealing*”, “*surface characterisation*”, “*scanning electron microscopy*”, “*profilometer*”, “*surface roughness*”, “*FTIR*”, “*Raman microspectroscopy*”, “*X-ray photoelectron spectroscopy (XPS)*”*,* “*energy dispersive X-ray spectroscopy (EDS)*”, “*bond strength*”, and “*fracture mode*”. Neither PRISMA guidelines were followed, nor PROSPERO registration was undertaken; no language restrictions were imposed.

The time frame for the search was from 1999, the year in which Pascal Magne and Douglas first introduced the IDS technique, until 2025. Eligible articles included both original research (in vitro*,* in vivo, and clinical trials) and review articles (systematic and narrative). No restriction was imposed on study design, provided that the article addressed aspects of IDS, dentine bonding, or surface/interface characterisation.

Titles and abstracts were initially screened to exclude studies unrelated to adhesive dentistry, restorative procedures, or dentine surface analysis. Full-text evaluation was conducted for all potentially relevant articles. Emphasis was placed on identifying studies that investigated the morphologic, topographic, or chemical characteristics of the sealed dentine surface either directly after IDS, after provisionalisation, or following mechanical/chemical surface reactivation before final cementation.

The included literature was then analysed and categorised according to methodology (e.g., bond strength testing, SEM analysis, spectroscopic techniques), focus (e.g., mechanical outcomes vs. surface analysis), and clinical stage of surface evaluation.

Duplicate records were removed by DOI/PMID matching and, where unavailable, by normalised title–author–year comparison; ambiguous cases were resolved by manual inspection. Database retrieval was complemented by backward and forward citation chaining to capture additional relevant studies beyond the initial queries. Stage-by-stage numerical tallies were not compiled, as a protocol-driven systematic workflow was not undertaken. Grey literature and non-English sources were considered at screening; however, inclusion was restricted to peer-reviewed items with extractable data.

No formal risk-of-bias scoring or study-level grading was undertaken, as the review was conceived as an exploratory, concept-driven mapping of the surface-characterisation gap.

## 3. Results

### 3.1. Clinical Rationale and Benefits of Immediate Dentine Sealing

#### 3.1.1. Bond Strength, Restoration Survival and Marginal Integrity

Immediate Dentine Sealing (IDS) enhances not only the adhesive strength of indirect restorations, but also their long-term marginal integrity and survival. Several studies have demonstrated superior bond strength outcomes, quantified as microtensile (µTBS) [19,20,21,22,23,24] and microshear (µSBS) [17,25,26] values, when IDS is applied compared with delayed dentine sealing protocols [11,27], particularly when combined with a flowable resin layer [24] that acts as a stress absorbing intermediary [11,19,28]. Moreover, fatigue resistance under cyclic loading has also been shown to improve with IDS [29,30]. Specimens restored with CAD/CAM ceramics like lithium disilicate and subjected to thermomechanical stress displayed fewer adhesive failures and greater fracture strength when an IDS protocol was employed [10,13,31]. Notably, a recent meta-analysis confirmed that IDS significantly augments fracture resistance of posterior indirect restorations, although material-dependent variability was observed [8,20].

Importantly, bond strength issues are intimately linked to marginal integrity. How well a restoration seals at the tooth–restoration interface profoundly impacts bacterial penetration, dye microleakage, staining, and ultimately restoration longevity. In molar inlays with gingival margins extending into dentine, IDS, whether employing total-etch or self-etch bonding systems, has been shown to significantly reduce microleakage following thermocycling [1,14,32]. Conversely, laboratory findings suggest that IDS does not always achieve complete enamel–dentinal sealing across all margins, indicating that optimal application technique and material selection remain critical [33]. Yet, CAD/CAM overlays cemented under IDS demonstrate notably improved marginal adaptation, with reduced marginal gaps (reported ~48–54 µm vs. ~79–87 µm in non IDS teeth), especially when dual cure or flowable resin cements are utilised [9,23].

Restoration survival also benefits from this enhanced marginal performance. Although clinical evidence is supportive, currently led by the 11-year prospective trial on ceramic laminate veneers [15], additional long-term randomised clinical trials are desirable. The improved seal is credited for preventing marginal staining, a known precursor of restoration failure, and protecting against fracture progression.

Overall, IDS exerts a multifaceted protective effect, manifested as increased adhesive interface strength (µTBS/µSBS), improved fatigue and fracture resistance under load, superior marginal adaptation with reduced microleakage and gap formation, diminished marginal staining and contamination risk, and enhanced long-term restoration survival and integrity. These cumulative benefits underscore the practical value of IDS in adhesive dentistry. Nonetheless, it is noteworthy that despite the mechanical and marginal advantages, none of the referenced studies evaluate the surface quality of the sealed dentine prior to cementation, a critical mechanistic link that justifies the targeted focus of this review.

#### 3.1.2. Postoperative Hypersensitivity and Pulpal Protection

One of the principal rationales for the clinical adoption of IDS lies in its capacity to mitigate postoperative dentinal hypersensitivity and to preserve pulpal health [4,8,14]. Tooth preparation results in the exposure of freshly cut dentine, rendering the pulpo-dentinal complex vulnerable to a range of irritants including thermal stimuli, bacterial ingress, and mechanical trauma. IDS offers an early and effective barrier, re-establishing dentinal sealing before provisionalisation [8,34]. Randomised clinical trials have consistently shown that IDS reduces hypersensitivity compared to delayed dentine sealing protocols [12]. A randomised controlled trial found that patients reported markedly lower visual analogue scale (VAS) scores when IDS was applied before the provisional phase, suggesting more comfortable healing and better short-term patient outcomes [35]. The reduction in sensitivity can be attributed to the immediate occlusion of dentinal tubules and the prevention of fluid movement in accordance with the hydrodynamic theory of pain transmission [36].

Additionally, a histological study supports the biocompatibility of IDS. More retailed, in an in vivo canine model, the findings indicated that IDS was associated with only a mild inflammatory response, and the integrity of the odontoblastic layer was largely preserved. No signs of pulpal necrosis or severe reaction were observed, providing reassurance regarding the histological safety of the procedure [37]. These results are of particular importance considering that many clinicians are hesitant to apply adhesive systems on freshly exposed dentine in close proximity to the pulp chamber.

From a clinical standpoint, the sealing of dentine prior to provisionalisation prevents bacterial contamination and the diffusion by-products from provisional materials, which are known to be insufficient barriers against oral fluids and microorganisms [14]. IDS thus serves not only as a biological safeguard but also enhances patient comfort by reducing stimuli-induced discomfort during the temporisation phase. The protective effect of IDS may also extend into the long term by preventing the chronic pulpal stress. IDS potentially reduces the incidence of post-restorative sensitivity and the need for endodontic intervention in susceptible cases [38].

Collectively, these findings substantiate the biological rationale for IDS as a technique that promotes pulpal preservation and significantly enhances patient comfort by attenuating postoperative hypersensitivity. In the context of adhesive indirect restorations, these effects further support its clinical integration as a standard preparatory procedure.

### 3.2. Analytical Characterisation of the Sealed Dentine Interface: Current Evidence and Limitations

#### 3.2.1. Techniques Used to Study the Dentine–Resin Interface After IDS

The current understanding of the dentine–resin interface formed through Immediate Dentine Sealing (IDS) is largely based on in vitro and in vivo studies that employ mechanical and microscopic analyses to assess adhesion, structural integrity, and failure modes. While informative, these approaches predominantly rely on cross-sectional or fracture-based methodologies, offering limited insight into the actual surface characteristics of sealed dentine prior to adhesive luting [2,14].

Microtensile bond strength (µTBS) testing remains the most widely used technique to evaluate the performance of adhesive strategies following IDS. Numerous studies included in this review utilised µTBS to compare etch-and-rinse versus self-etch systems, universal adhesives, or dual-curing luting agents [11,19,20,21,22,23,27,31,39,40,41,42]. SEM-based failure mode analysis is commonly used to complement μTBS data by identifying adhesive, cohesive, or mixed failure patterns [20,22,23,31,40,41,42,43].

Scanning electron microscopy (SEM) is frequently employed in IDS research, but its application is almost exclusively confined to cross-sectional imaging of fractured specimens or resin–dentine interfaces after µTBS or thermocycling [20]. These images provide valuable information on hybrid layer formation and resin tag penetration, but they do not reflect the morphology of the sealed dentine surface at the time of final bonding [44]. Top-down or surface SEM imaging is extremely scarce, resulting in a significant knowledge gap regarding the topography and structural integrity of the reactivated dentine surface [34]. In one study, surface SEM imaging was conducted on sealed dentine in order to examine the effects of pre-cementation cleaning [24]. In another investigation, different cleaning protocols were compared using similar imaging techniques to visualize the treated surfaces [17]. However, both studies were limited to secondary electron detection and did not include compositional or quantitative surface analysis.

Marginal integrity and microleakage assessments, typically performed through dye penetration or silver nitrate tracing, further confirm the sealing efficacy of IDS. However, these methods offer only indirect evidence regarding the condition of the bonding substrate after provisionalisation [32,45]. Similarly, studies assessing the effect of temporary materials on bond strength focus primarily on whether provisional cements impair adhesion, but do not analyse the post-cleaning surface itself [17,42].

Only a few isolated studies have addressed parameters such as permeability, hydrophilicity, or surface energy, and these factors are typically inferred from bond degradation or water storage effects rather than measured directly [41]. Although changes in adhesive layer thickness have been documented following air abrasion, diamond bur roughening, or ultrasonic cleaning prior to cementation [46], surface topography, roughness, and elemental distribution of the sealed dentine remain largely unexplored. In one study, surface morphology was examined using three dimensional surface analysis techniques; however, no quantitative parameters were extracted, and no statistical treatment of the data was provided [47].

Finally, it must be emphasised that even the limited surface analyses available rarely represent the clinical substrate at the time of definitive bonding. The surface is not merely the “sealed dentine” immediately post-adhesive application, nor the aged surface post-provisionalisation, but rather the reactivated dentine surface following mechanical cleaning, typically via air abrasion, pumice polishing, or diamond bur roughening [48,49]. Despite being the actual substrate subjected to adhesive luting, this reactivated dentine surface has seldom been directly examined using advanced surface-sensitive techniques.

#### 3.2.2. Surface Versus Cross-Sectional Characterisation

While much of the existing literature on Immediate Dentine Sealing (IDS) focuses on the assessment of bond strength and the integrity of the resin–dentine interface, the vast majority of these studies rely on cross-sectional or fracture-based methodologies. These include microtensile bond strength (µTBS), shear or push-out testing, and corresponding scanning electron microscopy (SEM) of fractured surfaces [10,13]. While informative, these methods focus mainly on the internal structure of the adhesive interface after it has failed, often overlooking the intact surface that actually bonds with the luting agent during clinical cementation [19,49,50].

Cross-sectional SEM and failure analysis provide insight into hybrid layer formation, adhesive tag morphology, and failure modes [31]. However, these approaches do not allow for the direct characterisation of the exposed sealed dentine surface, particularly after mechanical or chemical reactivation. Consequently, the functional state of the adhesive interface as it interacts with resin cements remains largely undocumented [48,51].

A recurring observation across numerous in vitro studies is the reliance on destructive sectioning followed by internal imaging. For instance, some studies demonstrated favourable effects of IDS on fracture patterns and bond durability using µTBS and fatigue testing, but none of these investigations involved surface-level morphological or chemical analyses prior to final bonding [4,26]. Even in studies involving thermocycling, artificial ageing, or interaction with provisional cements, the sealed surface is rarely imaged in situ [42].

Very few articles venture beyond these established methods. In isolated cases, the thickness of the cured adhesive layer and the effect of surface cleaning (e.g., air abrasion, ultrasonic cleaning, or pumice) were examined in relation to bond strength recovery [41,46]. However, even these contributions stop short of characterising how such treatments affect the topography, surface energy, or elemental composition of the sealed dentine. No information is provided on possible changes in smear morphology, adhesive microroughness, or chemical oxidation due to reactivation procedures, all of which may affect wetting, monomer infiltration, and final polymerisation [52].

Only rare exceptions explore qualitative imaging of the dentine surface after IDS. For instance, in a research comparing IDS and Er:YAG laser ablation as pretreatment options before inlay luting, yet their evaluation focused on bond performance without reporting the surface properties generated by either protocol [53]. Likewise, in a different experimental work where studied temporary cement removal protocols and found that air abrasion with glycine and D-limonene cleaning were effective in maintaining bond strength, but again, the morphology and chemistry of the cleaned surface were not investigated beyond performance metrics [17].

Similarly, an experimental work on surface cleaning after provisionalisation underlined the incomplete removal of temporary cement residues even after aggressive cleaning. Yet, although some SEM was used to detect residuals, no detailed surface characterisation or quantitative imaging was performed.

Advanced analytical techniques such as atomic force microscopy (AFM), surface optical non-contact profilometry, X-ray photoelectron spectroscopy (XPS), Raman microspectroscopy, ToF-SIMS are applied in a limited or qualitative manner in the reviewed IDS literature, despite their potential to provide crucial information about surface roughness, elemental distribution, polymer cross-linking, and hydrophilicity, all directly relevant to bonding outcomes [45,54].

The absence of top-down SEM or AFM analyses is particularly striking given that IDS-treated dentine surfaces are often mechanically reactivated just prior to final cementation. Whether this reactivation is performed using a fine-grit diamond, pumice slurry, or aluminium oxide particles, it produces a new surface that bears little resemblance to the initial sealed substrate. It is precisely this reactivated dentine surface, shaped by IDS and modified by cleaning or abrasion, that ultimately interacts with the resin luting agent. Yet, its physical and chemical characteristics remain virtually unreported [49,50].

Some attempts at molecular or nanoleakage characterization, such as the FE-SEM/EDS study, or the work on interfacial polymer networks, hint at the complexity of the adhesive interface [43,44]. However, these efforts are again limited to internal sections or to chemically stable conditions, with no reference to surface state dynamics following pre-cementation reactivation [43].

Taken together, current outcomes remain dominated by bond-strength surrogates and cross-sectional microscopy. Direct, non-destructive assessment of the reactivated dentine surface at cementation is infrequent and usually descriptive, limiting comparability and mechanistic inference. The implications of this limitation are addressed in the Discussion: Appendix A (Table A1) summarises the in vitro evidence and highlights studies with surface-level/top-down imaging.

### 3.3. Limitations in Current Characterisation

Despite substantial evidence for the clinical benefits of IDS, critical limitations persist in the characterisation of the sealed dentine substrate. Most investigations rely on destructive or cross-sectional read-outs (e.g., microtensile/micro-shear bond strength and fractographic SEM) that interrogate internal failure surfaces rather than the surface that actually receives the luting agent. Consequently, the reactivated external surface immediately prior to cementation, the functional bonding substrate in clinical practice, remains incompletely documented.

Clinically, the surface that receives the luting agent is neither the freshly sealed layer nor the surface merely aged during provisionalisation, but the mechanically reactivated dentine produced by air abrasion, pumice polishing, or diamond roughening [17]. This reactivated dentine surface has seldom been examined directly. Widely used read-outs—cross-sectional SEM, failure-mode analysis, and dye-based microleakage—interrogate internal sections or indirect surrogates and therefore do not characterise the surface attributes relevant at cementation (topography, roughness/wettability, elemental and molecular composition, surface energy) [27,42]. As a result, a disconnect persists between laboratory assessments of bonding and the clinical substrate present at cementation.

Furthermore, dentine adhesion is highly sensitive to structural and chemical substrate variations. Factors such as tubule orientation, dentine depth, hydration, the presence of sclerotic or caries-affected tissue, and surface energy have been shown to influence resin infiltration and hybridisation [49,50,51,54]. Although advanced characterisation has been widely used in dentine-bonding research, such approaches have seldom been applied specifically to IDS [44,52].

The IDS substrate is further complicated by sequential modification: initial sealing, potential contamination and ageing during provisionalisation, and mechanical reactivation prior to luting. Each phase can alter surface morphology, wettability, permeability, and chemical functionality [17,46]. However, there is no standardised methodology for documenting or controlling these dynamic surface states. Consequently, heterogeneity in experimental outcomes may arise not only from differences in adhesive protocols, but also from the undocumented bonding surface.

A number of studies have attempted to address this issue by evaluating cleaning protocols applied after provisionalisation, with bond strength measurements used as the primary outcome [17,47]. Stereomicroscopy has occasionally been used to assess contamination from resin-based temporaries, but without morphological or chemical surface analysis [42]. One available study used top-down SEM to visualise the reactivated dentine surface and reported differences associated with the presence of a flowable resin layer [24]. However, the findings were qualitative in nature and did not incorporate compositional, structural, or quantitative surface analysis. Collectively, these investigations highlight the clinical relevance of the reactivated dentine surface, while underscoring the lack of detailed surface-level characterisation in the existing IDS literature.

## 4. Discussion

The present review underscores a critical limitation in the scientific foundation of Immediate Dentine Sealing (IDS): the absence of systematic surface-level characterisation of the sealed dentine immediately prior to definitive cementation. Although IDS has consistently demonstrated clinical benefits, such as improved bond strength, enhanced fracture resistance, reduced hypersensitivity, and increased restoration longevity [10,11,46,53], the precise nature of the reactivated dentine surface remains only partially understood. While a limited number of studies have incorporated top-down imaging or surface profiling (as summarised in Table A1), integrated topographical and chemical surface analysis of the reactivated dentine surface is still lacking. This gap remains a key barrier to elucidating how IDS improves adhesive performance and to developing protocols or materials that might optimise it further.

As the primary objective was to delineate an under-characterised substrate and to articulate testable directions, a narrative synthesis was adopted rather than a protocol-driven systematic review. This approach may be more susceptible to selection bias and less comprehensive than preregistered systematic methodologies. Mitigation steps included explicit description of databases, date ranges and search terms; duplicate removal (DOI/PMID or normalised title–author–year); staged screening against predefined inclusion criteria; and citation snowballing. Stage-wise numerical tallies were not compiled because a protocol-driven systematic workflow was not undertaken; accordingly, claims of scarcity are qualified as under-representation within the peer-reviewed, indexed literature for the stated timeframe and criteria, and the conclusions should be interpreted as hypothesis-generating.

In clinical reality, the sealed dentine surface undergoes multiple transformations before final cementation: initial adhesive application, potential contamination and water sorption during provisionalisation, and mechanical reactivation, typically via diamond roughening, air abrasion, or pumice polishing [17,46]. As a result, this dynamically altered substrate differs both chemically and morphologically from the initially sealed surface. Yet its properties remain insufficiently defined. Although some studies have investigated cleaning or conditioning protocols, they rarely extend beyond bond strength and fracture mode read-outs. Only a limited number have examined the reactivated dentine surface using top-down approaches, such as secondary-electron SEM, surface profiling, or optical non-contact profilometry, and even then, methodological consistency and quantification are limited. Three-dimensionality has at times been claimed despite the underlying method relying on simplified two-dimensional traces without validated spatial metrics or statistical substantiation [47]. Likewise, SEM documentation is often restricted to representative areas without consistent imaging parameters or structured comparison across conditions [24]. Protocols seldom specify repeatable fields of view, depth control, magnification/calibration settings, or provide AFM-based nanoscale topography or wettability measurements. The lack of such procedural stringency hampers comparability and interpretability, underscoring the need for standardised, quantitative protocols to assess the morphological features and wettability of the reactivated dentine surface. Variability in specimen preparation and operator-dependent reactivation protocols (medium, pressure, time, grit) and heterogeneity in test modality and geometry (µSBS/SBS; bonded area; crosshead speed) are common, and surface-level verification of the reactivated dentine surface is often incomplete. These features limit cross-study comparability; accordingly, effect directions are emphasised and pooled magnitudes are avoided.

From this standpoint, surface-science descriptors of the reactivated dentine surface help explain why apparently similar reactivation protocols yield divergent adhesive outcomes. From a surface-science perspective, wettability (contact-angle behaviour) and the associated surface-free-energy components are expected to influence primer spreading and penetration into the conditioned smear, favouring a more continuous hybrid layer when contact angles are lower and the polar component is higher. Nanoscale roughness (e.g., AFM-derived Sa/Sq over defined scan sizes, with stated tip radius and set-point force) can increase effective interfacial area and, within practical bounds, facilitate micro-mechanical interlocking; excessive or non-uniform roughening, however, risks heterogeneous wetting and local stress concentrations. Chemical oxidation and related shifts in surface functional groups may modulate interfacial energy and co-polymerisation potential across resin–resin contacts; conversely, over-oxidation or residual reactive species could impair conversion. Taken together, these descriptors of the reactivated dentine surface offer a mechanistic rationale for observed differences in bond-strength trends and failure modes. Accordingly, a minimal reporting set is encouraged—static/dynamic contact angle with at least two probe liquids and derived surface-energy components (e.g., Owens–Wendt or vOCG), AFM roughness metrics (Sa/Sq) over calibrated areas with acquisition parameters, and, where relevant, a polymerisation index (e.g., FTIR-based degree of conversion) as a proxy for cross-linking—to enable interpretable links between surface state and clinical adhesive performance.

In parallel, the chemical composition of the reactivated dentine surface remains largely unexplored, further reinforcing the need to pair surface-sensitive measurements with clinical performance readouts. Surface-specific, chemically resolved analyses (e.g., EDS, XPS, FTIR microscopic imaging, Raman microspectroscopy, ToF-SIMS) could probe elemental distribution, functional-group chemistry, oxidation or oxygen-inhibited layers, and surface contaminants; complementary compositional contrast from backscattered-electron (BSE) imaging can further aid interpretation. However, their application in IDS-related research has been sparse and largely qualitative, with many reports omitting such analyses altogether or relying on bulk surrogates inferred from bond strength, fracture mode, or ageing effects. This gap limits mechanistic inference on how surface treatments and ageing alter the bonding potential of reactivated dentine.

While surface-sensitive techniques are well positioned to characterise the reactivated dentine surface, their use on moist, heterogeneous substrates faces practical constraints, moisture sensitivity, limited and potentially unrepresentative fields of view, and beam-related artefacts (e.g., charging), as well as cost, availability and training. Pragmatic mitigations include controlled specimen conditioning, low-vacuum/cryo or ‘top-down’ modes where available, and non-destructive proxies (optical profilometry with calibrated filtering; standardised contact-angle protocols), combined with triangulation across complementary methods. Brief reporting of surface conditioning, area selection, vacuum/temperature and acquisition parameters can enhance reproducibility without expanding the present review into a technical treatise.

Further limitation arises from studies that report bond strength and failure modes without concomitant interface characterisation, i.e., without describing the IDS-treated dentine, the reactivated dentine surface state, or its interaction with specific adhesive systems. For example, IDS effectiveness has been attributed primarily to functional monomers [55] following pumice/rotary-brush cleaning, while overlooking differences in filler content between formulations, despite separate evidence that filler reinforcement is decisive for predictable IDS performance [24]. Comparable bond strength outcomes have at times been interpreted through divergent hypotheses [22,47], and some protocols deviate substantially from standard IDS practice (e.g., comparing IDS with self-adhesive cements without dentine bonding steps or surface characterisation, mixing filled and unfilled adhesives, or applying sandblasting for 10 s on flat dentine that plausibly removes most of the unfilled adhesive layer), thereby confounding interpretation [39]. Where “surface roughness” has been discussed from secondary-electron images alone [25], the analysis under-utilises available analytical modalities. In the absence of substrate-specific protocols and chemically resolved surface analyses, drawing generalisable conclusions remains problematic.

In conclusion, while IDS confers well-documented clinical benefits, a more detailed understanding of the reactivated dentine surface is essential to consolidate its scientific foundation. Bridging the gap between surface science and adhesive dentistry will refine current techniques. It will also pave the way for innovations in adhesive systems explicitly engineered for this complex, yet critically important substrate. Although some interpretations attribute IDS effectiveness to co-polymerisation with a partially polymerised or oxygen-inhibited layer. However, such a mechanism is unlikely to persist after provisionalisation and surface reactivation. In routine clinical practice, reactivation procedures, air abrasion, pumice polishing, or rotary diamond roughening are not standardised for time, pressure, angulation, or instrument selection. So, the outer adhesive layer may differentially be removed, partially re-exposing the underlying dentine. Accordingly, the bonding surface at the time of cementation may no longer consist of aged resin but of morphologically and biologically favourable dentine, including regions of intertubular dentine and resin-infiltrated tubules, which support the formation of a renewed hybrid layer. Variability in bond strength across cleaning methods and adhesive systems further suggests that the quality of the reactivated dentine surface may be more decisive than the intrinsic chemistry of the initial adhesive. This perspective situates IDS within a substrate-driven paradigm in which the key determinant of clinical success is not the residual reactivity of a cured polymer but the nature of the selectively reactivated dentine surface.

## 5. Conclusions

Immediate Dentine Sealing (IDS) is a clinically validated strategy that improves adhesion and reduces post-operative sensitivity in indirect restorations. Although its mechanical and biological benefits are well supported, the characteristics of the sealed dentine, particularly after provisionalisation and reactivation, lack surface-specific definition. While a few studies have incorporated top-down imaging or surface profiling, systematic, high-resolution morphological and chemical analyses are still lacking. Addressing this gap through advanced, surface-sensitive methodologies could lead to more predictable adhesive protocols and improved restorative outcomes.

## 6. Future Directions

Future investigations should shift focus from internal, fractured interfaces to the external, reactivated dentine surface that directly interacts with the luting agent. This necessitates the design of experimental protocols that systematically characterise the reactivated dentine surface following IDS using surface-sensitive, non-destructive techniques such as atomic force microscopy (AFM), optical non-contact profilometry, Raman microspectroscopy, X-ray photoelectron spectroscopy (XPS), top-down SEM, and ToF-SIMS.

Despite some preliminary efforts, the literature still lacks quantitative and chemically resolved data that accurately depict the nature of the bonding substrate at the time of cementation. Mapping surface morphology, roughness parameters, and compositional gradients, particularly after common reactivation procedures such as air abrasion or diamond bur roughening, would provide critical insights into the underlying adhesion mechanisms.

Moreover, the development of adhesive systems specifically tailored to interact with this dynamically modified surface could improve clinical outcomes and protocol predictability. Standardising surface characterisation methodologies would further enhance reproducibility across studies and reduce inter-study variability (Table 1). In practical terms, standardised reporting, quantitative surface metrics, and multivariate integration of mechanical with surface/chemical datasets are encouraged. Bridging this gap is essential to move beyond empirically guided bonding strategies towards a substrate-informed, evidence-based paradigm in adhesive dentistry.

## Figures and Tables

**Table 1:** IDS stages (Initial sealing → Provisionalisation → Reactivation → Cementation) with indicative levels of characterisation and analytical techniques per phase. The listed techniques are illustrative, not exhaustive; selection and combinations should be tailored to the study question, resources, and validation requirements, with emphasis on the reactivated dentine surface prior to cementation. AFM, atomic force microscopy; contact angle (static/dynamic); FTIR-ATR, Fourier-transform infrared spectroscopy with attenuated total reflectance; µSBS, microshear bond strength; µTBS, microtensile bond strength; Ra, arithmetic mean roughness; Sa/Sq, areal roughness average/root-mean-square (AFM/profilometry); SFE, surface free energy; SEM, scanning electron microscopy; ToF-SIMS, time-of-flight secondary ion mass spectrometry; XPS, X-ray photoelectron spectroscopy.

	Initial Sealing	Provisionalisation	Reactivation	Cementation
Level of Characterisation	- Chemical composition - Surface topography - Wettability	- Drift in wettability/surface free energy - Topographic change (swelling/deposits) - Contaminant uptake from temporaries/biofilm	- Drift in wettability (contact angle/SFE) - Roughness/texture (nano–micro scale) - Surface chemistry/oxidation (functional groups)	- Bond strength - Fracture mode - Microleakage
Indicative analytical techniques	- FTIR-ATR/Raman (degree of conversion/functional groups) - AFM or optical profilometry (Sa/Sq or Ra) - Contact angle (≥2 probe liquids) + SFE (*optional: XPS if available*)	- Contact angle/SFE (before vs. after ageing) - AFM/profilometry (swelling, deposits) - ToF-SIMS or Raman (residuals/contaminants)	- Report medium/pressure/time/grit (standardised) - AFM/profilometry (calibrated area; stated parameters) - XPS/Raman (oxidation/functional groups) - Contact angle (≥2 liquids) + SFE	- µTBS/µSBS (defined bonded area; units) - Cross-sectional SEM /fractography - Microleakage (dye/fluorescent tracer or fluid infiltration)

## Data Availability

The original contributions presented in this study are included in the article. Further inquiries can be directed to the corresponding author.

## Appendix A

**Table A1:** Overview of in vitro studies investigating Immediate Dentine Sealing (IDS), including the surface reactivation protocols, measured parameters, and analytical techniques employed. Studies marked with an asterisk (*) included direct top-down imaging or surface profiling of the sealed dentine substrate. (SEM: Scanning Electron Microscopy, UTM: Universal Testing Machine, FM: Failure Mode, μTBS: micro-Tensile Bond Strength, TBS: Tensile Bond Strength, μSBS: micro-Shear Bond Strength, SBS: Shear Bond Strength, ATR-FTIR: Attenuated Total Reflectance Fourier Transform Infrared Spectroscopy, μCT: micro-Computed Tomography).

Study Reference	Surface Reactivation	Measured Parameters	Instrumentation/Methods
M.M. Stavridakis et al., 2005 [46]	Al_2_O_3_ or prophylaxis paste	adhesive layer thickness	SEM
R. Osorio et al., 2005 [31]	Not specified	μTBS, FM, microleakage	UTM, SEM, thermocycling, load-cycling
P. Magne et al., 2005 [20]	CoJet	μTBS, FM	UTM, SEM
R.M. Duarte et al., 2006 [28]	Not specified	TBS, FM morphological characterisation of the hybrid layer	UTM, SEM
* F. Falkensammer et al., 2014 [47]	pumice or sandblasted with silica-coated Al_2_O_3_ or glycin, or CaCO_3_	SBS, FM, adhesive layer thickness, roughness	UTM, stereomicroscope, profilometry
* M. Özcan & S. Lamperti, 2015 [25]	50-μm Al_2_O_3_ or 30-μm SiO_2_ (various pressures), or prophylaxis paste, or pumice	μSBS, FM	UTM, SEM
M.M. Gresnigt et al., 2016 [11]	pumice/CoJet/Silanisation	fracture strength, FM	Thermocycling (10,000× cycles), static loading (1 mm/min).
* C.J. Ribeiro da Silva et al., 2016 [42]	pumice	μTBS, FM, top-down imaging	UTM, optical microscope, SEM
C.R.G. van den Breemer et al., 2017 [30]	pumice/CoJet/Silanizing	artificially ageing, Failure under compression, FM, Marginal adaptation	chewing simulator, thermocycling, SEM
V.C. Brigagão et al., 2017 [27]	pumice	μTBS, FM	UTM, stereomicroscopy
* D. Augusti et al., 2018 [17]	Hand scaler or sandblasted with Al_2_O_3_ or Glycine powder or D-Limonene chemical solvent	μSBS, FM	UTM, stereomicroscopy, SEM top-down imaging
RC Ferreira-Filho, 2018 [39]	Not specified	μTBS, FM	UTM, stereomicroscope, SEM
N.G.L. Hironaka, 2018 [41]	pumice	μTBS, FM, Dentine-cement interface—transition zone	UTM, SEM, Raman spectroscopy
C.R.G. van den Breemer et al., 2019 [19]	pumice or pumice/Cojet	μTBS, FM	Microtensile, stereomicroscopy
S.A.G. Bilal Utku et al., 2020 [26]	Not specified	SBS, cross-section imaging	UTM, SEM
H.F.A. Gailani et al., 2021 [40]	sandblasting with CaCO_3_/new adhesive layer	μTBS, FM	UTM, stereomicroscopy, SEM
O.M. Sakr, 2021 [53]	Not specified	SBS	UTM
A. Abdou et al., 2021 [23]	alcohol	μTBS, bonding resin DC%, adhesive layer thickness, Cross-section	UTM, ATR-FTIR, SEM
* M.A. de Carvalho et al., 2021 [24]	pumice prior to impressions. Al_2_O_3_/H_3_PO_4_ prior to cementation	μTBS, FM	UTM, SEM, Top-down imaging after pre-delivery cleaning
N. Saadeddin et al., 2022 [10]	pumice/CoJet/H_3_PO_4_	Fracture Strength, FM	thermocycling, UTM (compressive loads), stereomicroscopy
N. Pheerarangsikul et al., 2022 [55]	pumice	SBS, FM	UTM, SEM
T. Kovalsky et al., 2022 [56]	sandblasting	adhesive layer thickness, cross-section imaging	Optical microscopy and SEM
E.A.E. Abo-Alazm et al., 2022 [21]	nan	μTBS, Dentine permeability	UTM, fluid filtration
B. Mueller et al., 2023 [29]	pumice/Al_2_O_3_/H_3_PO_4_	Fatigue strength, Survival rate, FM	thermocycling and accelerated fatigue tests stereomicroscopy SEM
A. Krishnan et al., 2025 [32]	Not specified	Pull-out bond strength, Microleakage	UTM, thermocycling and sectioning
R.Q. Ramos et al., 2025 [22]	sandblasting/H_3_PO_4_	μTBS, FM, Interface characterisation	UTM, chewing simulator, SEM, light microscopy, μCT
